# Delayed Recovery After Deep Brain Stimulation Surgery for Parkinson's Disease Under General Anesthesia-Cases Report

**DOI:** 10.3389/fsurg.2022.811337

**Published:** 2022-03-01

**Authors:** Long Feng, Yaohong Liu, Hao Tang, Zhipei Ling, Longhe Xu, Weixiu Yuan, Zeguo Feng

**Affiliations:** ^1^Department of Anesthesiology, Hainan Hospital of Chinese People's Liberation Army (PLA) General Hospital, Sanya, China; ^2^Department of Neurosurgery, Hainan Hospital of Chinese People's Liberation Army (PLA) General Hospital, Sanya, China; ^3^Department of Anesthesiology, The Third Medical Center of Chinese People's Liberation Army (PLA) General Hospital, Beijing, China; ^4^Department of Pain, The First Medical Center of Chinese People's Liberation Army (PLA) General Hospital, Beijing, China

**Keywords:** delayed awakening, deep brain stimulation, Parkinson's disease, general anesthesia, propofol

## Abstract

**Objective:**

Parkinson's disease (PD) is a neurodegenerative syndrome, and deep-brain stimulation (DBS) is an effective therapy for carefully screened patients with PD. However, delayed recovery after anesthesia, which occurs after taking prolonged general anesthesia for such patients, has been reported less frequently in literature. This report explores the possible causes of postoperative awakening delay in patients undergoing DBS surgery due to general anesthesia and provides a reference for anesthesia management of similar operations in the future.

**Case Presentation:**

Three patients with PD elective underwent DBS surgery. The first patients demonstrated walking disability, gait deficits, unstable posture, limb stiffness, and imbalance. The second demonstrated left limb static tremor, stiffness, and bradykinesia. The third demonstrated bradykinesia, rigidity, walking deficits, and decreased facial expression. These included two males and one female with a mean patient age of 60.7 ± 6.7year, weight of 63.7 ± 11 kg, the height of 163.3 ± 7.6 cm, and preoperative American Society of Anesthesiology rating of 2.3 ± 0.6. The preoperative Glasgow Coma Scale mean score was 15. All patients completed the operation under general anesthesia (the mean anesthesia time was 5.3 ± 1.1 h). The mean operation time was 252 ± 60 min. The mean bleeding volume was 50 ml, and the urine volume was 867 ± 569 ml. However, all the patients showed unconsciousness after 95 ± 22 min after stopping the anesthetic, and the respiratory function was in good condition, but they could not cooperate with anesthesiologists and had no response to the anesthesiologist's instructions. The mean hospital stay was 17 ± 7 days. All patients were discharged uneventfully. The average number of days patients followed up postoperatively was 171 ± 28.5 days. Motor and speech were improved significantly postoperatively in three patients compared with preoperatively. Taking anti-Parkinson medication was markedly reduced. There were no complications during postoperative follow-up.

**Conclusions:**

To prevent delayed recovery occurring after DBS surgery in Parkinson's disease, it is recommended to take scalp nerve block + general anesthesia to complete the procedure while avoiding general anesthesia.

## Background

Parkinson's disease is a neurodegenerative syndrome involving multiple motor and non-motor symptoms. DBS is an effective therapy for carefully screened patients with PD who have to disable on-off fluctuations, dyskinesia, and medication-resistant tremor (1). Sleep disturbances are common in PD and are related to medication effect, tremor, painful dystonia, and nocturia leading to difficulty with sleep initiation, fragmentation of sleep, restless legs syndrome, and rapid eye movement behavior disorder, which all negatively impact sleep and quality of life (2, 3). Both the Subthalamic-nucleus (STN) and Globus Pallidus Interna (GPi) have been shown to influence the interaction between wakefulness and sleep, particularly, which are more likely to impact rapid eye movement sleep (4).

General anesthesia (GA) with tracheal intubation is the routine procedure during the second stage of DBS operation, which is the implantation of the pulse generator. The recent Meta-analysis indicated that there were no significant differences between the GA and Local Anesthesia groups in operation time, and the incidence of adverse events (including postoperative speech disturbance and intracranial hemorrhage) in DBS surgery (5). However, the literature related to DBS anesthesia mainly reports the safety, advantage, and disadvantages of different anesthesia methods, the effects of different anesthetic drugs on intraoperative microelectrode recording (MER), and the perioperative precautions of anesthesiologists (6–8). Anesthetic agents (such as benzodiazepines, barbiturates, propofol, etomidate, and volatile agents) affect the background spontaneous firing and the neuronal spike activity patterns of basal ganglia nuclei (9, 10), mainly through activation of gamma-aminobutyric acid (GABA) receptors. However, to our knowledge, few studies have summarized and reported the causes of postoperative awakening delay in DBS patients.

Therefore, this report aimed to explore the possible causes of postoperative awakening delay in patients undergoing DBS surgery due to GA and to provide a reference for anesthesia management of similar operations in the future. Based on our clinical experience of these problems, we posit that the delay of postoperative recovery caused by DBS operation under GA may result from many factors.

## Case Presentation

A 55-year-old man, a 59-year-old man, and a 68-year-old woman had a diagnosis of PD, and received elective DBS procedures for bilateral STN brain electrode implantation under GA in the neurosurgery department of our hospital with the diagnosis of PD.

**Figure 1:**
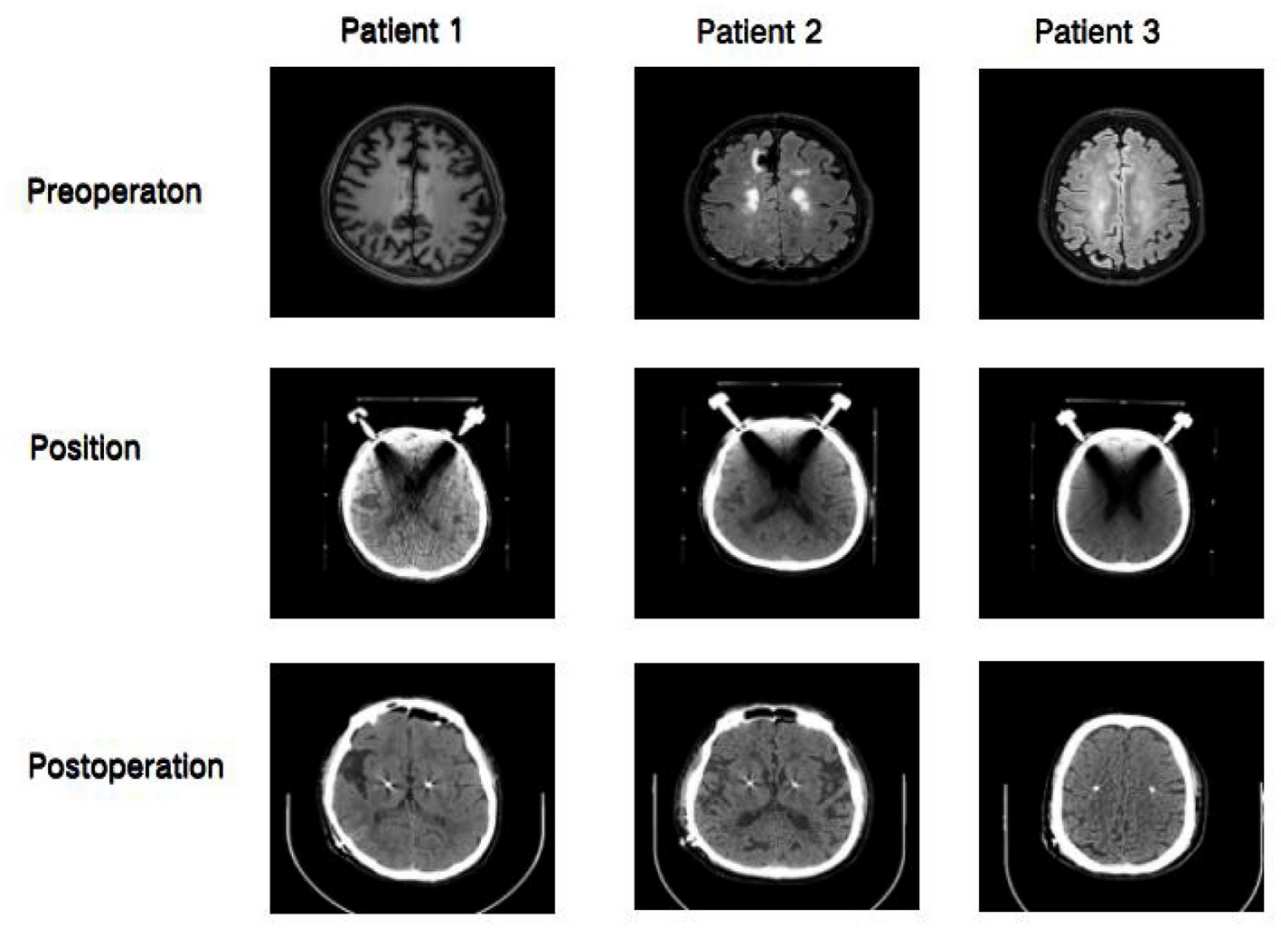
Preoperative, electrode localization, and postoperative brain CT. Three patients underwent brain CT before operation, electrode positioning and after operation. The patient was admitted to the hospital for preoperative examination of the head CT (Preoperation); Before surgery upper head frame positioning CT (Positon); After surgery to determine the electrode position CT (Postoperation;).

The first 55-year-old man demonstrated walking disability, gait deficits, unstable posture, limb stiffness, and imbalance. The PD symptoms could be ameliorated after Levodopa administration. Along with the disease progress, the efficacy of Levodopa was diminished. In addition, the patient suffered from type 2 diabetes and a history of meningioma surgery. The patient had previously undergone surgery at a local hospital for meningioma, but the specific situation is unknown. The preoperative Glasgow Coma Scale (GCS) score was 15, and the ability of orientation, comprehension, judgment, calculation, short-term and long-term memory were normal. Both hands rotation test, finger nose test, and bilateral heel knee tibia test were normal. The difficulty of standing sign cannot be completed, and the straight-line walking test could not be completed. The muscle strength of the upper limb and lower limb was V levels, and muscle tension is normal. The sinus heart rate was 62 beats/min, and no clear abnormalities in the chest radiograph. The cardiac ultrasound ejection fraction was 61%. There was no special abnormality in the preoperative laboratory examination (Table 1). The preoperative cranial magnetic resonance was normal (Figure 1). To localize the optimal response site to intraoperative microstimulation, levodopa medications were discontinued the night before surgery.

**Table 1:** Laboratory examination indicators before and after surgery of all patients.

**Characteristics**	**Normal rang**	**Patients 1**		**Patients 2**		**Patients 3**	
		**Preoperation**	**postoperation**	**Preoperation**	**postoperation**	**Preoperation**	**postoperation**
Hemoglobin (g/L)	Male: 137–179 Female: 116–155	155	149	143	143	118	112
Hematocrit (L/L)	Male: 0.4–0.52 Female: 0.37–0.47	0.451	0.445	0.416	0.410	0.349	0.330
Red blood cell (10^12^/L)	Male: 4.3–5.9 Female: 3.9–5.2	4.81	4.60	4.93	4.93	3.49	3.82
White blood cell (10^9^/L)	3.5–10.0	8.64	10.85	6.40	9.05	7.45	9.25
Neutrophils (10^9^/L)	0.05–0.07	0.768	0.785	6.40	9.05	7.45	9.25
Lymphocytes (10^9^/L)	0.20–0.40	0.152	0.129	0.291	0.123	0.250	0.148
Monocytes (10^9^/L)	0.03–0.08	0.061	0.075	0.064	0.065	0.048	0.048
Eosinophils (10^9^/L)	0.01–0.05	0.014	0.007	0.008	0.004	0.023	0.002
Basophils (10^9^/L)	0.00–0.01	0.005	0.004	0.005	0.002	0.005	0.003
Platelet count (10^9^/L)	100–300	213	215	221	191	264	230
Sodium (mmol/L)	130–150	143.5	141.8	143.7	139.0	141.2	138.6
Potassium (mmol/L)	3.5–5.5	4.08	3.87	3.87	4.00	4.03	4.02
Chloride (mmol/L)	94–110	102	103.4	102.4	98.7	102.6	101.2
Carbon dioxide (mmol/L)	20.2–30.0	24	20.9	25.1	20.9	24	47
Creatinine (μmol/L)	30–110	85	80	74	60	58	47
Urea nitrogen (μmol/L)	1.8–7.5	5.2	7.4	5.2	3.4	5.2	5.0

The second 59-year-old man demonstrated left limb static tremor, stiffness, and bradykinesia. The patient had a good response to Levodopa and dopaminergic promotor. However, the efficacy was decreased gradually. The patient had type B hepatitis for 20 years. A lacunar infarction was found for 6 years. The patient had previously undergone surgery at a local hospital for meningioma, but the specific situation is unknown. The preoperative GCS score was 15, and the ability of orientation, comprehension, judgment, calculation, short-term and long-term memory were normal. Both hands rotation test, finger nose test, and bilateral heel knee tibia test were normal. The closed eyes were difficult to stand up normally, and the straight-line walking test could not be completed. The muscle strength of the upper limb and lower limb was V levels, and muscle tension was enhanced. The ECG showed the ST-T changes and the sinus heart rate was 72 beats/min. The chest radiograph showed no significant abnormality, and the preoperative laboratory examination showed no special abnormalities (Table 1). Preoperative cranial magnetic resonance revealed multiple ischemic foci in the centrum semiovale (Figure 1). To localize the optimal response site to intraoperative microstimulation, Levodopa and dopaminergic promotor were discontinued the night before surgery.

The third 68-year-old woman presented with bradykinesia, rigidity, walking deficits, and decreased facial expression. The non-motor symptom included constipation. In addition, the mild cognitive deficits were appreciated, including decreased orientation, comprehension, calculation, and judgment. The patient had a good response to Levodopa and dopaminergic promotor. The efficacy decreased over time. Regarding the history, the patient suffered from hypertension for more than 15 years and type 2 diabetes for more than 23 years. The preoperative GCS score was 15 points, including decreased orientation, comprehension, judgment, calculation, short-term and long-term memory. The results showed that the hand rotation test was clumsy, the finger nose test was inaccurate, the bilateral heel knee tibia test could not be completed. It was difficult to close eyes, the standing sign examination was unstable, and the straight-line walking test could not be completed. The impaired coordination was noted. The muscle strength of the upper limb and lower limb is V levels, and the muscle tension was gear-like. The ECG sinus heart rate was 72 beats/min. The echocardiographic ejection fraction was 64%. The chest radiograph and the preoperative laboratory examinations showed no significant abnormalities (Table 1). The preoperative cranial magnetic resonance was normal (Figure 1). To localize the optimal response site to intraoperative microstimulation, Levodopa and dopaminergic promotor were discontinued the night before surgery.

## Anesthesia Medication and Program

All patients were routinely monitored for non-invasive blood pressure, electrocardiogram, and pulse oxygen saturation after entering the operation room. GA was conducted by tracheal intubation through an oral visual laryngoscope. The induction medication included: methylprednisolone 40 mg, midazolam 1–2 mg, etomidate 0.1–0.2 mg/kg, and/or propofol 0.5–1 mg/kg, rocuronium ammonium 0.5–0.6 mg/kg, sufentanil 0.2–0.3 μg/kg. All patients underwent continuous invasive radial artery pressure monitoring after intubation. Intravenous inhalation was combined with maintenance anesthesia (Sevoflurane 1–2%+ Propofol 1–2 mg/kg.h+Remifentanil 5–7 μg/kg.h). Intraoperative fluids were sodium acetate Ringer's solution and hydroxyethyl starch 130/0.4.

## Results

All patients completed the operation under GA. The mean anesthesia time and operation time was 5.3 ± 1.1 h, and 252 ± 60 min, respectively. The mean bleeding volume was 50 ml, and the urine volume was 867 ± 569 ml. Infusion volume was Crystalline solution 2,100 ± 500 ml and colloid 333 ± 289 ml. All patients did not have a blood transfusion. After the operation, the tracheal tube was successfully pulled out and returned to the ward.

All three patients showed unconsciousness after 95 ± 22 min after stopping the anesthetic. The respiratory function was in good condition, but they could not cooperate with anesthesiologists and had no response to the anesthesiologist's instructions (Table 2). Two of the patients showed apathy after the operation with their eyes closed, and there was no response to calls. On the other hand, one patient appeared to be indifferent, staring, and unresponsive to external calls. The first patient was suspected of a cerebral hemorrhage or cerebral infarction and subjected to a CT scan with a tracheal tube. However, the CT scan suggested normal changes after surgery. The other two patients showed normal condition in the delayed CT examination. The patients were sent back to the ward with continuous monitoring, and all patients recovered their preoperative state after 2–4 h. None of the patients underwent secondary tracheal intubation or were sent to the intensive care unit for treatment. None of the patients had brain hemorrhage or cerebral infarction after re-examination of brain CT (Figure 1).

**Table 2:** Perioperative volume and length of stay.

**Characteristics**	**Patient 1**	**Patient 2**	**Patient3**	**Mean value**
Operation time (min)	248	195	314	252 ± 60
Blood loss (ml)	50	50	50	50
Urine (ml)	1,500	700	400	867 ± 569
Transfusion (ml)	0	0	0	0
Infusion (ml)				
Crystalline	2,600	2,100	1,600	2,100 ± 500
Colloid	500	500	0	333 ± 289
ASA rank	II	II	III	2.3 ± 0.6
Anesthesia-time (h)	5.45	6.28	4.18	5.3 ± 1.1
Recovery-time (min)	120	87	79	95 ± 22
In-hospital (day)	22	21	9	17 ± 7
Follow-up time (day)	142	199	171	171 ± 28.5

All patients recovered well and were discharged smoothly, with an average hospital stay of 17 ± 7 days. The average number of days patients were followed up postoperatively was 171 ± 28.5 days. Motor and speech were improved significantly postoperatively in three patients compared with preoperatively. Taking anti-Parkinson medication was markedly reduced. There were no complications during postoperative follow-up (Table 2).

## Discussion

DBS surgery is an effective treatment for patients with Parkinson's disease (11–13). Delayed awakening from anesthesia remains one of the biggest challenges for the anesthesiologist. The consciousness should be usually restored 60–90 min after stopping the anesthetic even in a long-term operation (11). The selection of anesthetic agents should have the least impact on microelectrode recordings. Zelcer et al. (12) indicates that in 443 patients with GA, the incidence of regaining consciousness beyond 90 min after the surgery was 9.46%. Misal et al. (13) also demonstrate that the risk factors responsible for delayed emergence from anesthesia include the patient factors, drug factors, surgical and anesthetic factors, and metabolic factors. In the early stage of this kind of operation in our hospital, the placement of the intracranial electrode and subcutaneous pacemaker were completed under GA. There have been frequent occurrences of delayed awakening after the operation. No intracranial hemorrhage was found in cranial CT. Because of the above clinical problems, we then changed the GA to scalp nerve block combined with GA, and no patient had delayed awakening. Unfortunately, there were still a number of patients (movement disorder) who could not tolerate DBS surgery while being awake or scalp nerve block. GA should be an appropriate alternative in clinical practice.

The following reasons may be the causes for delayed recovery after anesthesia in these patients.

Firstly, preoperation abrupt withdrawal of medications leads to parkinsonism-hyperpyrexia syndrome (PHS). This syndrome may occur after the DBS procedure for bilateral STN electrode placement. All patients usually stopped taking anti-Parkinson's oral medications before surgery in this report. One patient during the postoperative follow-up declaimed the perception of the doctor's communication, but it was unable to respond to the instructions. Kim et al. (14) also report that a 66-year-old female patient develops consciousness disturbance, high fever, hypercardiac, and hypertension after the DBS operation. PHS should be considered if the patient demonstrates changes in consciousness with hyperpyrexia after surgery, which cannot be explained by other reasons. PHS should be considered and adequate treatment should be given immediately to prevent death (14).

Secondly, the electrode possibly damages the surrounding brain tissue and/or lesion, affecting the peripheral thalamus during surgery. The previous study indicated that underlying pathophysiology of cognitive changes may be related to the contact stimulation on the DBS lead, pulse width of stimulation, frequency of stimulation, changes in medication doses, micro-lesion effect, laterality of DBS implantation, or target stimulated for therapy (1, 15). A recent study has reported that stimulating the central lateral nucleus of the thalamus can wake up anesthetized macaques to a certain extent (16). If the nucleus around the thalamus is injured during DBS surgery, it may affect the normal recovery of anesthesia after surgery. In a case report by Singh et al. (17), a patient with bilateral STN insertion for PD underwent laparoscopic cholecystectomy under GA. During reversal of anesthesia, the patient did not arouse. However, at this time, the DBS system was reactivated, followed by a sudden increase in entropy values and spontaneous eye Opening. Besides, there are two case reports of patients with an implanted neurostimulator who suffered from serious brain injuries due to heat generation at the tip of the DBS electrodes (18).

Thirdly, anesthetics and anesthesia depth may affect Electroencephalogram signal transduction. Many neurosurgeons prefer to avoid sedation in PD patients undergoing DBS placement, because some anesthetic drugs may abolish MER and symptoms (19). Raz et al. (10) found that propofol (50 μg/kg·min) can significantly decrease spiking and background electrical activity, as well as the root mean square power in the STN when recordings from the same target nucleus and coordinates were compared. Anesthetic agents (such as benzodiazepines, barbiturates, propofol, etomidate, and volatile agents) affect the background spontaneous firing and the neuronal spike activity patterns of basal ganglia nuclei, mainly through activation of GABA receptors (9, 10). Propofol is the most commonly used agent in DBS and has been shown to cause a global depression in neuronal discharge (7). Animal studies have shown that this effect is at least partially mediated by GABA receptor activation (20). These three patients with the delayed awakening are all operated on under propofol intravenous anesthesia, and the average operation time is 252 ± 60 min. If the intraoperative use of propofol causes inhibition of the above-mentioned brain electrical activity and neurotransmitter activity. Then high-frequency and strong stimulation may be used to determine the brain electrical signal when judging the electrode position during the operation. Venkatraghavan et al. (7) recommended that all anesthetic agents be turned off at least 15 min prior to MER and left off until electrode stimulation testing is completed. The delayed emergence may be connected with the depression of the previously affected ventral pallidal inputs to the septohippocampal system that mediates GA and awareness (21). However, Martinez-Simon et al. (19) quantify the effects of Dexmedetomidine and Propofol on basal ganglia neuronal activity by measuring LFPs during the DBS surgery. They find that the Dexmedetomidine (0.2 μg/kg·min) does not show a negative effect on local field potentials compared with propofol recording (19). Incremental doses of propofol significantly decline the deep brain activity, especially in the beta frequency range. Furthermore, the latest research found that scalp nerve block + GA is more suitable for this kind of surgery than regional or GA (22, 23). It can effectively avoid the influence of anesthetic drugs and depth on electroencephalogram signals. At the same time, it can also evaluate the degree of muscle habenula remission.

Lastly, intracranial gas embolism and hemorrhage may also affect the patient's recovery after surgery. One of the three patients had intracranial gas after surgery in Koi and colleagues' report (24). They found that among 371 patients, pneumocephalus are noted in 66% of wake DBS patients and 15.6% of asleep DBS patients. It is well-known that larger volumes of intracranial air have been correlated to the shifting of brain structures and impact the accuracy of electrode placement during DBS procedures (24). In this report, one patient had a small amount of intracranial pneumatosis, and no intracerebral hemorrhage was found in all patients by postoperative brain CT.

This study has some limitations. First, we report on only three patients in this cases report, meaning the sample size was small. Secondly, according to our clinical experience and relevant literature, we made a multivariate inference analysis on the delay of anesthesia awakening after DBS, but the exact reason remains to be confirmed by large samples and randomized controlled clinical studies.

In conclusion, for Parkinson's DBS implantation surgery under GA, the patient should be wary of delayed recovery from anesthesia. Firstly, the operation procedures under the whole course GA should be largely avoided, except for patients who cannot tolerate local anesthesia. Secondly, Dexmedetomidine is recommended during the operation and propofol can be considered as an alternative. Finally, for patients who have stopped oral medication before surgery, if there is a change in consciousness after surgery, were alert to the occurrence of PHS. In short, this delayed recovery after anesthesia is mainly characterized by the patient's indifference, inability to cooperate, and normal respiratory function. The patient will return to normal consciousness 2–4 h after the end of anesthesia and generally do not need special treatment.

## Data Availability Statement

The raw data supporting the conclusions of this article will be made available by the authors, without undue reservation.

## Ethics Statement

The studies involving human participants were reviewed and approved by the Ethical Committee of the Chinese People's Liberation Army General Hospital. Written informed consent was not provided because this is a retrospective analysis of clinical cases. Consent has been obtained with the patient and his family. At the same time, all information does not involve the patient's sensitive information and privacy. Written informed consent was obtained from the individual(s) for the publication of any potentially identifiable images or data included in this article.

## Author Contributions

WY, LX, ZF, and ZL contributed to the design of the study and the review of the literature. LF, HT, and YL participated in data collection and analysis and drifting of the manuscript. All authors have read and approved the manuscript.

## Conflict of Interest

The authors declare that the research was conducted in the absence of any commercial or financial relationships that could be construed as a potential conflict of interest.

## Publisher's Note

All claims expressed in this article are solely those of the authors and do not necessarily represent those of their affiliated organizations, or those of the publisher, the editors and the reviewers. Any product that may be evaluated in this article, or claim that may be made by its manufacturer, is not guaranteed or endorsed by the publisher.
